# Hearing ability is not a risk factor for admission to aged residential care of older persons in New Zealand

**DOI:** 10.1038/s41598-019-53457-y

**Published:** 2019-11-21

**Authors:** Philip J. Schluter, Megan J. McAuliffe, Deborah A. Askew, Hamish A. Jamieson

**Affiliations:** 1 School of Health Sciences, University of Canterbury – Te Whare Wānanga o Waitaha, Christchurch, New Zealand; 2 School of Clinical Medicine, Primary Care Clinical Unit, The University of Queensland, Brisbane, Australia; 3 School of Psychology, Speech and Hearing, University of Canterbury – Te Whare Wānanga o Waitaha, Christchurch, New Zealand; 4 New Zealand Institute of Language, Brain and Behaviour, University of Canterbury – Te Whare Wānanga o Waitaha, Christchurch, New Zealand; 5 Southern Queensland Centre of Excellence in Aboriginal and Torres Strait Islander Primary Health Care, Queensland Health, Inala, Queensland Australia; 6 Department of Medicine, University of Otago, Christchurch, Christchurch New Zealand; 7 Older Person’s Health, Canterbury District Health Board, Christchurch, New Zealand

**Keywords:** Outcomes research, Epidemiology

## Abstract

Aged residential care (ARC) admission needs are increasing beyond the available capacity in many countries, including New Zealand. Therefore, identifying modifiable factors which may prevent or delay ARC admissions is of international importance. Hearing impairment is common among older adults and thought to be an important predictor, although the current evidence-base is equivocal. Using the largest national database to date, competing-risk regression analysis was undertaken on 34,277 older adults having standardised home care assessments between 1 July 2012 and 31 May 2014, aged ≥65 years, and still living in the community 30 days after that assessment. Minimal hearing difficulty was reported by 10,125 (29.5%) participants, moderate difficulty by 5,046 (14.7%), severe difficulty/no hearing by 1,334 (3.9%), while 17,769 (51.8%) participants reported adequate hearing. By 30 June 2014, the study end-point, 6,389 (18.6%) participants had an ARC admission while 6,082 (17.7%) had died. In unadjusted competing-risk regression analyses, treating death as a competing event, hearing ability was significantly associated with ARC admission (p < 0.001). However, in adjusted analyses, this relationship was completely confounded by other variables (p = 0.67). This finding implies that screening for hearing loss among community-living older adults is unlikely to impact on ARC admission rates.

## Introduction

Globally, and within New Zealand, an uncharted demographic phenomenon is occurring; the accelerating ageing of populations through declining fertility and increasing life expectancies^1^. This demographic shift poses serious challenges across multiple sectors, including health and aged residential care (ARC) services, through rapidly increasing age-related chronic disease and condition numbers. So much so that the current approach to health and disability services provision is considered unsustainable in New Zealand^2,3^, as it has been elsewhere^4^. Integrated health systems centred on enabling people to stay in their own homes longer, with an interconnected structure supporting them to live well and take greater responsibility for their own health, has been mooted as one solution^4–6^. Among the principal envisioned downstream impacts of this strategy is a reduction in ARC entry and hospital admission demand^7^. At the heart of this integrated strategy is an emphasis on prevention, efficaciously targeting and ameliorating modifiable risk factors. One potentially modifiable risk factor, hearing impairment, is common among older people and may benefit from such an approach.

Hearing loss can have profound effects on individuals, not only on interpersonal communication, but also on health, independence, wellbeing, and quality-of-life^8^. In a 2018 New Zealand hearing loss prevalence study, conducted on 16,080 nationally representative participants, 23.5% of those aged 65–74 years and 39.4% of those aged ≥75 years reported impairment^9^. Similarly, 19.5% and 36.7% of adults aged 65–74 years and ≥75 years, respectively, reported hearing impairment across the United Kingdom, Germany and France in 2018^10^. Of the 50 million Americans aged >65 years, approximately 35% have hearing loss which is sufficient to make them hearing-aid candidates^11^. Despite hearing loss being ranked as the fifth leading cause of years lived with disability^12^, unmet treatment need remains high – even in developed countries such as the USA^8,13^. Yet some forms of hearing loss are preventable^14^, and many other forms are readily treatable^11^.

Hearing impairment is more frequent among people within ARC facilities than their age-standardised community counterparts, and opined to be an important antecedent disability which contributes to admission^15^. In a longitudinal study investigating predictors of institutionalization among adults age ≥75 years, those having mild hearing loss had increased odds of institutionalization estimated to be 2.2 (95% confidence interval [CI]: 1.1, 4.3) compared to those without hearing loss, while people with severe/profound hearing loss had estimated odds of 9.4 (95% CI: 2.1, 42.3)^16^. However, these findings have not been replicated elsewhere. In an unadjusted analysis, Evans and colleagues identified a statistically significant relationship between hearing impairment and ARC admission, which disappeared after adjustment^17^. Another study failed to find a statistically significant unadjusted relationship in their non-disabled, community-living sample of adults aged ≥70 years^18^. These equivocal findings are likely due to important study design and analytical differences^19^. Also, the competing risk of death has not been explicitly considered before – a factor which is germane to older populations where the likelihood of death is non-ignorable^20,21^.

Using current service delivery models projections, the required sector bed numbers within New Zealand ARC facilities needs to increase by 78% to 110% to accommodate the increase in extra residents and replacement of ageing facilities by 2026^2^. However, there are insufficient financial returns to support building new capacity and replacing ageing stock^2^. To mitigate this economic and resource burden, mutable factors contributing to ARC admission need to be identified and attenuated. Although many forms of hearing impairment are readily treatable^11^, it has received limited research attention with respect to ARC admission; meta-analyses and reviews often fail to identify or consider hearing loss within their scope^19,22^. Using the largest ever population dataset to date, this study seeks to determine whether hearing loss is an independent predictor of ARC admission for those aged ≥65 years, after controlling for a suite of confounders and employing apposite statistical methods that adjust for the competing event of death.

## Methods

### Study design

A time-to-event study following a national cohort.

### Participants

Home-based people age ≥65 years in New Zealand having an interRAI-HC assessment undertaken between 1 July 2012 and 31 May 2014, inclusive, and who consented to their data being used for planning and research purposes. Those who were admitted to ARC or died within 30 days of their assessment were excluded.

### Primary measures

The interRAI-HC instrument is used for all community care assessments on older people needing publicly funded long-term community services or ARC in New Zealand^23^. The interRAI-HC assessment form version 9.1 (© interRAI Corporation, Washington, D.C., 1994–2009), modified with permission for New Zealand, is used under license to the Ministry of Health (www.interrai.co.nz). It is comprised of 236 questions over multiple health and social domains, which form 27 standardized instruments, and yields internationally valid and reliable scales^23,24^. Hearing was elicited by asking respondents about their ability to hear (with hearing aid normally used), with response options: *adequate* – no difficulty in normal conversation, social interaction, listening to TV; *minimal difficulty* – difficulty in some environments (e.g., when person speaks softly or is more than 2 metres away); *moderate difficulty* – problem hearing normal conversation, requires quiet setting to hear well; *severe difficulty* – difficulty in all situations (e.g., speaker has to talk loudly or speak very slowly, or person reports that all speech is mumbled); and, *no hearing*. InterRAI-HC information is stored electronically and is National Health Index (NHI) number-linked, using encryption for data security^23^. The NHI is a unique identifier that is assigned to every person who uses health and disability support services in New Zealand.

ARC entry status and date of entry data were obtained from the Ministry of Health’s Contracted Care Payment System (CPSS) database. The CPSS contains all people who are publicly funded for such care (approximately 80% of all residents). However, CPSS also contains information on approximately 50% of those who are self-funding, as this is voluntarily entered by providers. Hence the CPSS database contains data on approximately 90% of those entering ARC^25^. At the time of data release, the CPSS database was complete and finalised for ARC information collected on or before the 30 June 2014, so this was assigned as the study end date. For those with an interRAI-HC assessment and a subsequent ARC admission captured within the CPSS database, ARC status was indicated and study time was equated to the difference between the ARC admission and interRAI-HC assessment dates.

Survival status and date of death data were extracted from the National Mortality Collection Register, also held by the Ministry of Health^26^. A cut-off date of 30 June 2014 was also applied, to ensure complete capture of registered deaths and consistency with the CPSS dataset. For those with an interRAI-HC assessment whose death was subsequently captured within the National Mortality Collection Register and who had not admitted to an ARC, death was indicated and study time was equated to the difference between death and interRAI-HC assessment dates. Note, death after an ARC admission was not relevant for this analysis – as it occurred after the event of interest. Eligible participants without an ARC admission and who survived beyond the study end date were defined as being censored, with study times equal to 30 June 2014 minus their interRAI-HC assessment dates.

### Demographic and potentially confounding measures

A suite of demographics and potentially confounding variables have been associated with ARC admission. Consistent with previous published studies and clinical experience, the following variables were utilized: age; sex; ethnicity; marital status; living arrangements; body mass index (BMI); activities of daily living (ADL); instrumental ADL (IADL), excluding phone use; alcohol consumption; smoking status; vision status; depression; delirium; history of falls; recent hospitalisation; diagnoses of stroke/cerebrovascular accident (CVA), chronic obstructive pulmonary disease (COPD), cancer, congestive heart failure (CHF), and coronary heart disease (CHD); fatigue; timed 4 m walk; urinary incontinence; and, self-rated health. All these measures arose from the interRAI-HC assessment, and the variable definition and classification details appear in the supplementary materials.

### Procedure

A detailed account of the interRAI-HC assessment instrument and procedure within New Zealand has been described previously^23^. In brief, the standardised interRAI-HC instrument is used for the conduct of all community care assessments on older people needing publicly funded long-term community services or ARC. Individuals are referred by a health practitioner to have their needs assessed by one of the more than 1,800 trained interRAI-HC assessors. Assessors visit clients in their own home to produce individualized care-plans according to a standardized protocol. Participants are explicitly asked if they consent to their de-identified interRAI-HC information being used for planning and research purposes; approximately 93% of participants provide this consent^23^. Where an individual had more than one interRAI-HC assessment between 1 July 2012 and 31 May 2014, only the first assessment was utilized.

### Data management

Three separate national databases were interrogated and research datasets created, each containing encrypted NHI numbers for their respective members; encryption was undertaken by the Ministry of Health as an additional layer of identity protection. These databases included the: (i) interRAI-HC; (ii) CPSS; and, (iii) National Mortality Collection Register. The interRAI-HC research dataset contained information on all people who had at least one interRAI-HC assessment between 1 July 2012 and 31 May 2014 and consented to their data being used for planning and research purposes. Importantly here, the CPSS research dataset contained the date of entry for those admitted to ARC facilities, while the National Mortality Collection Register research dataset contained the date of death for those who had died. These latter two databases were known to have complete information on or before the 30 June 2014, thus this was assigned as the study end date.

All data were extracted by the Ministry of Health, using the interRAI-HC dataset membership as the index. This was done by extracting NHI numbers for all consenting members between 1 July 2012 and 31 May 2014 from this database, and then separately deterministically linking them to the CPSS and National Mortality Collection Register databases. When a NHI match was found, then this populated its respective research dataset, together with the date of admission or death whichever applicable. People changing ARC facilities have multiple admission dates; in these instances the first date was used. The interRAI-HC, CPSS and National Mortality Collection Register research datasets were securely released to the research team, deterministically matched by participants’ encrypted NHI numbers^23^, exclusion criteria applied, and then subjected to pursuant statistical analyses.

### Statistical analysis

Reporting of analyses were informed by the RECORD guidelines^27^. Analyses employed competing-risk regression models, using the Fine and Grey method^28^, and treated ARC admission as the primary event of interest and death as the competing event. Both unadjusted and fully adjusted models were undertaken. Rather than using the bivariable analyses to screen risk factors or potential confounders, in the spirit of Sun and colleagues^29^, all candidate variables were included in the multivariable model regardless of their statistical significance. Sub-hazard ratios (SHRs) and associated 95% CIs were reported, and Wald’s type III χ^2^ statistic used to determine a variable’s significance.

To gauge the extent of confounding explained between hearing ability and ARC admission, a forward stepwise selection process was undertaken with the hearing ability status variable forced into the model. At each step, the variable combination which minimized the Bayes information criterion (BIC) was selected^30^, and the Wald’s type III χ^2^ statistic associated with the hearing ability variable calculated. To establish whether there was a likely causal relationship, rather than simply associative, stratified interaction terms were assessed and reported^31^. Spearman’s ρ was employed to measure the correlation between ordinal variables.

To assess the contribution of the hearing ability status variable in the adjusted model, Harrell’s *c*-statistic was employed^32^. Models are typically considered reasonable when the *c*-statistic >0.7 and strong when >0.8^33^. To derive the *c*-statistic, the sample was randomly partitioned into two datasets of equal size; the first used as a training dataset to fit the model, and the second used as a test dataset to make prediction assessments^32^. All analyses and graphics were performed using Stata SE version 15.1 (StataCorp, College Station, TX, USA), and α = 0.05 defined statistical significance.

### Ethics

Clearance for this study and its protocol was approved by the Ministry of Health’s Health and Disability Ethics Committee (14/STH/140). All methods were performed in accordance with that Ethics Committee’s relevant guidelines and regulations. The study only included those participants who provided written and informed consent to their data being used for planning and research purposes. The Ministry of Health does not release data to researchers for those who do not provide this consent. As this investigation is a secondary analysis of routine collected de-identified data, written and informed consent was not obtained for this specific study nor was it deemed necessary in the Ethics Committee’s approval. The research databases released by the Ministry of Health contained participants’ encrypted NHI numbers, needed for matching, but has all personally identifying information removed. The encryption code is held by the Ministry of Health and no other.

## Results

### Sample description

Overall, 47,257 individuals had an interRAI-HC assessment between 1 July 2012 and 30 June 2014. Of these, 2,797 were aged <65 years, 1,907 had their assessment after 31 May 2014, and 8,276 were admitted to ARC or had died within 30 days of their assessment, and so were excluded from the study. This left an eligible sample of 34,277 people, with an average age of 82.1 years (range: 65, 105 years). Their demographic profiles are presented in Table 1.

**Table 1:** Demographics of eligible participants at study inception (n = 34,277), and partitioned by outcome at the study end date (30 June 2014).

	At study inception	Outcome at study end date					
		Remained at home		Admitted to ARC		Died	
	n	n	(%)	n	(%)	n	(%)
***Age group (years)***							
65–74	5,804	4,184	(72.1)	756	(13.0)	864	(14.9)
75–84	14,323	9,475	(66.2)	2,534	(17.7)	2,314	(16.2)
85–94	13,088	7,644	(58.4)	2,852	(21.8)	2,592	(19.8)
95+	1,062	503	(47.4)	247	(23.3)	312	(29.4)
***Sex***^**a**^							
Female	21,334	14,222	(66.7)	3,905	(18.3)	3,207	(15.0)
Male	12,942	7,583	(58.6)	2,484	(19.2)	2,875	(22.2)
***Ethnicity***							
European	30,254	18,955	(62.7)	5,964	(19.7)	5,335	(17.6)
Māori	1,782	1,189	(66.7)	192	(10.8)	401	(22.5)
Pacific	1,214	910	(75.0)	97	(8.0)	207	(17.1)
Other	1,027	752	(73.2)	136	(13.2)	139	(13.5)
***Marital status***							
Married/civil union/de facto	13,519	8,649	(64.0)	2,396	(17.7)	2,474	(18.3)
Widowed	16,794	10,577	(63.0)	3,233	(19.3)	2,984	(17.8)
Divorced/separated	2,330	1,558	(66.9)	436	(18.7)	336	(14.4)
Never married	1,388	863	(62.2)	286	(20.6)	239	(17.2)
Other	246	159	(64.6)	38	(15.4)	49	(19.9)
***Residential arrangements: living with***^**a**^							
Spouse/partner only	10,893	6,991	(64.2)	1,925	(17.7)	1,977	(18.1)
Spouse/partner & other(s)	1,237	804	(65.0)	182	(14.7)	251	(20.3)
Alone	16,606	10,830	(65.2)	3,213	(19.3)	2,563	(15.4)
Child (not spouse/partner)	3,625	2,245	(61.9)	621	(17.1)	759	(20.9)
Other relative(s)	847	534	(63.0)	136	(16.1)	177	(20.9)
Non-relative(s)	1,068	402	(37.6)	311	(29.1)	355	(33.2)

Note: ^a^1 observation missing.

### Admission to aged residential care and mortality outcome patterns

By 30 June 2014, 6,389 (18.6%) participants had been admitted to ARC, 6,082 (17.7%) had died without entering such care, and 21,806 (63.6%) remained at home. The median (Q_1_, Q_3_) follow-up times were 4.6 months (2.4, 8.4 months), 6.2 months (3.0, 12.0 months), and 9.4 (5.0, 13.9 months) for each outcome, respectively. In total, this equated to 25,168.5 person-years participants were at risk and under observation. Of the 6,389 participants admitted to ARC, 2,545 (39.8%) subsequently died prior to the study end-point (30 June 2014). The demographic breakdown of participants by the study outcomes is also contained within Table 1, and differences were observed. Compared to Europeans, Māori and Pacific older adults were less likely to have ARC admissions; participants residing with non-relatives were more likely to have ARC admissions or die compared to those living with relatives; and, participants aged ≥ 95 years were more likely to die than their younger counterparts.

### Hearing ability

Hearing ability data were available from all but three participants. Only 53 (0.2%) participants were classified as having no hearing, and thus were combined with those having severe difficulty hearing. The distribution of hearing ability is presented in Table 2. Overall, 51.8% of participants had adequate hearing, 29.5% had minimal difficulty, 14.7% had moderate difficulty, while the remaining 3.9% had severe hearing difficulty or no hearing. From Table 2, a monotonically increasing pattern in the percentage of participants with ARC admission and reduced hearing ability can be observed. Overall, 17.4% of those with adequate hearing ability at assessment were subsequently admitted to ARC whereas 24.5% of those with severe difficulty or no hearing were admitted. A similar relationship can be observed between hearing ability and death.

**Table 2:** Distribution of hearing ability by outcome at the study’s end date, together with sub-hazard ratio (SHR) and 95% confidence interval (CI) estimates for the unadjusted and adjusted (using complete cases n = 33,993; 99.2% of sample) competing-risk regression analyses for aged residential care (ARC) admission.

Hearing ability^b^	Remained at home		ARC admission		Died		Unadjusted		Adjusted^a^	
	n	(%)	n	(%)	n	(%)	SHR	(95% CI)	SHR	(95% CI)
Adequate	11,852	(66.7)	3,088	(17.4)	2,829	(15.9)	1	(reference)	1	(reference)
Minimal difficulty	6,367	(62.9)	1,926	(19.0)	1,832	(18.1)	1.10	(1.04, 1.17)	0.99	(0.94, 1.06)
Moderate difficulty	2,928	(58.0)	1,048	(20.8)	1,070	(21.2)	1.21	(1.13, 1.30)	0.96	(0.89, 1.03)
Severe difficulty/no hearing	657	(49.3)	327	(24.5)	350	(26.2)	1.42	(1.27, 1.59)	1.00	(0.88, 1.12)

Note: ^a^adjusted for age group, sex, ethnicity, marital status, living arrangements, BMI, ADL, IADL, alcohol consumption, smoking status, vision status, depression, delirium, history of falls, recent hospitalisation, stroke/CVA, COPD, cancer, fatigue, congestive heart failure, coronary heart disease, timed 4 m walk, urinary incontinence, and self-rated health; ^b^3 observations missing.

### Unadjusted analyses

**Figure 1:**
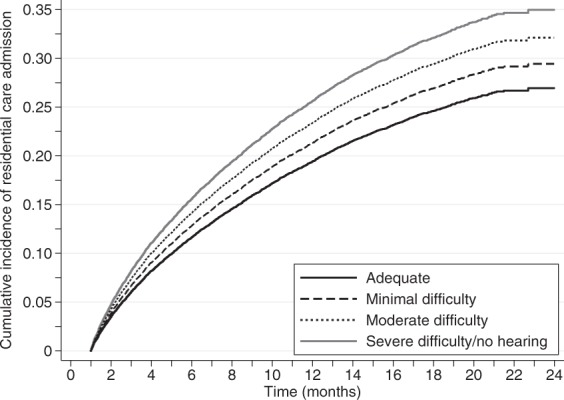
Cumulative incidence of admission to aged residential care (ARC) by level of hearing ability.

Applying competing-risk regression models, treating death as a competing event, hearing ability was significantly associated with ARC admission (p < 0.001), and the likelihood of admission appeared to be dependent on hearing ability status; see Table 2 and Fig. 1. In Fig. 1, the cumulative incidence of ARC admission curve associated with each hearing ability classification appeared to be importantly different from the others. This was confirmed using pair-wise post hoc tests from the unadjusted competing-risk regression model, with ARC admission rates higher for those with minimal hearing difficulty compared to those with adequate hearing (p = 0.001), for those with moderate hearing difficulty compared to those with minimal difficulty (p = 0.02), and for those with severe hearing difficulty or no hearing compared to those with moderate difficulty (p = 0.01).

### Adjusted analyses

Tables 2 and S1 (in the supplementary material) includes the results from the full multivariable competing-risk regression model, which included n = 33,993 (99.2%) participants. (Table S1 gives the distribution of the demographic and potentially confounding variables, together with their associated unadjusted and adjusted SHR and 95% CI estimates.) In this adjusted model, hearing ability was no longer significant (p = 0.67). In an effort to understand which variables explained the observed significant unadjusted relationship, a forward stepwise selection process was undertaken. Table 3 presents the resultant findings, together with the SHR estimates and associated 95% CIs. With the addition of just two variables in the competing-risk regression model, namely age and IADL, the association between hearing ability status and ARC admission became non-significant (p = 0.81).

**Table 3:** Sub-hazard ratio (SHR) and associated 95% confidence interval (CI) estimates for hearing ability status at each step in the forward stepwise competing-risk regression model that selects variables which minimizes the Bayesian information criterion (BIC) from all demographic and potentially confounding factors, together with the associated Wald’s type III χ^2^ statistic for the significance of the hearing ability status variable.

Model	Variables	Adequate		Minimal difficulty		Moderate difficulty		Severe difficulty/no hearing		BIC	Wald’s type III χ^2^ statistic	
		SHR	(95% CI)	SHR	(95% CI)	SHR	(95% CI)	SHR	(95% CI)		χ_3_^2^	p
M_0_=	Hearing status	1	(reference)	1.10	(1.04, 1.17)	1.21	(1.13, 1.30)	1.42	(1.27, 1.59)	125,855.6	57.7	<0.001
M_1_=	M_0_ + IADL	1	(reference)	1.07	(1.01, 1.31)	1.07	(1.00, 1.15)	1.15	(1.02, 1.29)	124,947.2	9.98	0.02
M_2_=	M_1_ + age group	1	(reference)	1.01	(0.95, 1.07)	0.98	(0.91, 1.05)	1.03	(0.92, 1.16)	124,826.1	0.95	0.81

The relationship between hearing impairment and IADL was further investigated. Spearman’s ρ = 0.13 for their correlation; a level typically regarded as weak. The competing-risk regression model which included hearing impairment, age and IADL, together with the interaction between hearing impairment and IADL, yielded the SHR and associated 95% CI estimates contained within Table 4. This interaction was non-significant (*p* = 0.17), as was the main effect term associated with hearing impairment (*p* = 0.12). Within each IADL stratum, all the SHR estimates for hearing impairment yielded 95% CIs which included the null value, whereas the pattern of SHR estimates increased similarly and monotonically for IADL categories between the hearing impairment strata – and had overlapping 95% CIs (see Table 4).

**Table 4:** Estimated sub-hazard ratios (SHRs) and associated 95% confidence intervals (CIs) for the interaction between hearing ability and instrumental activities of daily living (IADL), adjusted for age, overall and by the strata specific groups.

	Hearing ability								SHR for minimal difficulty within strata of IADL		SHR for moderate difficulty within strata of IADL		SHR for severe difficulty/no hearing within strata of IADL	
	Adequate		Minimal difficulty		Moderate difficulty		Severe difficulty/no hearing							
	SHR	(95% CI)	SHR	(95% CI)	SHR	(95% CI)	SHR	(95% CI)	SHR	(95% CI)	SHR	(95% CI)	SHR	(95% CI)
**IADL**														
Q_1_ (0–14)	1.00	(reference)	1.17	(0.99, 1.37)	1.19	(0.94, 1.50)	1.40	(0.87, 2.26)	1.17	(0.99, 1.37)	1.19	(0.94, 1.50)	1.40	(0.87, 2.26)
Q_2_ (15–24)	1.87	(1.66, 2.12)	1.97	(1.72, 2.26)	1.87	(1.57, 2.22)	2.06	(1.50, 2.83)	1.05	(0.93, 1.19)	1.00	(0.85, 1.17)	1.10	(0.80, 1.50)
Q_3_ (25–33)	2.72	(2.42, 3.05)	2.60	(2.29, 2.95)	2.75	(2.38, 3.19)	3.06	(2.45, 3.82)	0.96	(0.86, 1.06)	1.01	(0.89, 1.15)	1.12	(0.91, 1.39)
Q_4_ (34–42)	3.44	(3.07, 3.86)	3.33	(2.94, 3.77)	3.07	(2.67, 3.52)	3.17	(2.64, 3.80)	0.97	(0.88, 1.07)	0.89	(0.79, 1.00)	0.92	(0.78, 1.08)
**SHR for IADL within strata of hearing ability**														
Q_2_ (15–24)	1.87	(1.66, 2.12)	1.69	(1.45, 1.98)	1.57	(1.22, 2.02)	1.47	(0.84, 2.56)						
Q_3_ (25–33)	2.72	(2.42, 3.05)	2.23	(1.92, 2.59)	2.31	(1.82, 2.93)	2.18	(1.31, 3.62)						
Q_4_ (34–42)	3.44	(3.07, 3.86)	2.86	(2.46, 3.31)	2.57	(2.04, 3.24)	2.26	(1.38, 3.69)						

Finally, the predictive power of the full multivariable model, fitted on a training dataset and calculated on the test dataset, yielded a Harrell’s *c*-statistic of 0.6811 (95% CI: 0.6714, 0.6907); not importantly different from that when hearing ability status was excluded with 0.6810 (95% CI: 0.6713, 0.6706; p = 0.49). This represents better than chance prediction but less than the threshold (of 0.7) for a model demonstrating ‘reasonable’ predictive power.

## Discussion

Consistent with previous literature^15–17^, a strong and significant relationship between hearing impairment and ARC admission was initially identified in this study. For instance, those with severe hearing difficult or no hearing had odds of ARC admission 1.42 (95% CI: 1.27, 1.59) that of their counterparts with adequate hearing. However, like that identified by Evans and colleagues^17^, this relationship was fully explained by other variables – so that those with severe hearing difficult or no hearing had odds of ARC admission 1.00 (95% CI: 0.88, 1.12) that of their counterparts with adequate hearing in the adjusted analyses. In predicting ARC admission, the inclusion of age and IADL into the statistical model rendered hearing impairment as being non-significant (p = 0.81) in its contribution, completely confounding the significant unadjusted relationship. Here, IADL excluded phone use, as it might be considered a direct marker for hearing loss, but included: meal preparation; ordinary housework; managing finances; managing medications; stairs use; shopping; and, transportation (see the supplementary materials). By design, IADLs measure basic functional necessities, no matter where one lives, and typically represent the first areas requiring outside support. This functional assessment tool allows for the monitoring of older adults across the health continuum, from relative independence through to episodes of care^34^.

It has been recognised that IADL is associated with both hearing impairment^35^ and ARC admission^36^, and it might also be argued that it lies on the causal pathway between hearing impairment and ARC admission. Informed by the recommendations of Knol and VanderWeele^31^, stratum-specific SHRs were presented. It was evident from these analyses that hearing impairment was not differentially affected by IADL level, and that its non-significant relationship with ARC admission within each IADL group was consistent across all the IADL quartiles. In contrast, the patterns observed between IADL groups and ARC admission within the hearing impairment groups were similar. If hearing impairment was causally related to ARC admission, a different non-significant pattern would be expected within at least one IADL stratum. Finally, it might be argued that the hearing ability and IADL were largely explaining a similar health domain. However, the weak correlation between these variables makes it difficult to support this notion.

Predictively, it is noteworthy that the final adjusted model remained relatively weak, with Harrell’s *c*-statistic < 0.7. This implies that other important unmeasured variables exist in predicting ARC admission beyond a particular individual’s interRAI-HC profile, such as family, community, geographic, economic, and political driving forces^2^. Notwithstanding, in the final adjusted model, several other potentially mutable factors appear and deserve further investigation, including vision impairment and depression.

This large, national, contemporary study derived from a standardised instruments with broad suite of variables and few missing values, administered by a trained workforce has a number of strengths^23^. The large number of person-years, high uptake of participants consenting to the use of their data, ability to deterministically link national datasets through encrypted NHIs, and the use of apposite biostatistical methods in competing-risk regression analyses and prediction all add to the study’s strength and robustness^20^. However, the study is not without limitations. Arguably, the primary weakness centres on the variable of interest; hearing impairment was not determined through comprehensive audiological assessment. Nonetheless, clear differential patterns emerged in the unadjusted analyses and the question itself has strong face validity – which adds strength to this measure. Moreover, this hearing measure has been successfully employed elsewhere^37,38^. Another potential weakness is that hearing impairment is likely to be time-varying, yet only a baseline assessment was used here. However, if the purpose is to identify modifiable factors for intervention, then intrinsically this is not problematic but serves to reduce the predictive capacity of the final adjusted model. Another limitation is that the CPSS database only contains approximately 50% of participants who privately fund their ARC admission; which accounts for around 10% of all older adults entering care^25^. This 10% is likely to have different demographic and socio-economic profiles compared to the 90% contained within the CPSS database, but the extent and consequence of these differences is unknown. Moreover, the 30-days exclusion criterion applied within this study was designed to identify and remove older adults with acute needs or terminal illnesses, and the interRAI-HC assessment was simply a vehicle to expedite ARC admission. Although employed elsewhere^21,25^, this period remains arbitrary and the potential for misclassification exists.

Although not found to be statistically related to ARC admission, hearing loss among older adults is common and unmet treatment remains high^8,13^. Future research establishing the validity of the interRAI-HC hearing impairment variable against audiologically assessed standardised measures would strengthen the robustness of this evidence, as would the replication of this study’s findings in other geographically dispersed populations. Furthermore, future research aimed at developing models which improve ARC admission prediction power, through identification of additional confounders and reduction of measurement variability, is also encouraged. Particularly if these additional confounders are potentially modifiable, and amenable to change. Given the high unmet treatment need, yet detrimental sequelae of hearing loss, it would benefit from routine screening in primary health care for older adults^39^. Those suffering hearing loss should have treatment options discussed, managed, and be repeatedly assessed. Ultimately, this is likely to both enhance older adults’ health and wellbeing, and significantly reduce the deleterious effects of hearing loss among their families and society at large.

## Conclusions

While hearing impairment is often underserved in the older population, and there are compelling reasons why some have suggested that hearing loss may be an antecedent to ARC admission, no evidence was found here to support this supposition. The increased likelihood of ARC admission for those with hearing impairments was completely explained by other factors. While a null finding, it has been argued that replication studies are valuable and, indeed, keeps science on track^40^. Especially when the current evidence-base is of mixed quality and producing equivocal findings. As hearing loss does not appear to be an independent predictive antecedent to ARC admission, screening for hearing is unlikely to impact on admission rates.

## Supplementary information

Supplementary materials for: Hearing ability is not a risk factor for admission to aged residential care of older persons in New Zealand

## Data Availability

The datasets used for statistical analysis are held by New Zealand’s Ministry of Health. Application to use these data must be made through this Ministry.
