# Supplementary material for: Hearing ability is not a risk factor for admission to aged residential care of older persons in New Zealand
Source: Sci Rep. 2019 Nov 21;9:17272. doi: 10.1038/s41598-019-53457-y (PMC6872587; doi:10.1038/s41598-019-53457-y)
Supplement: Supplementary file 1 — Supplementary materials for: Hearing ability is not a risk factor for admission to aged residential care of older persons in New Zealand [file 41598_2019_53457_MOESM1_ESM.docx]

**Supplementary materials for:**

**Hearing ability is not a risk factor for admission to aged residential care of older persons in New Zealand**

Philip J. Schluter*^1,2^, Megan J. McAuliffe^3,4^, Deborah A. Askew^2,5^, Hamish A. Jamieson^6,7^

1. School of Health Sciences, University of Canterbury – Te Whare Wānanga o Waitaha, Christchurch, New Zealand
2. School of Clinical Medicine, Primary Care Clinical Unit, The University of Queensland, Brisbane, Australia
3. School of Psychology, Speech and Hearing, University of Canterbury – Te Whare Wānanga o Waitaha, Christchurch, New Zealand
4. New Zealand Institute of Language, Brain and Behaviour, University of Canterbury – Te Whare Wānanga o Waitaha, Christchurch, New Zealand
5. Southern Queensland Centre of Excellence in Aboriginal and Torres Strait Islander Primary Health Care, Queensland Health, Inala, Queensland, Australia
6. Department of Medicine, University of Otago, Christchurch, Christchurch, New Zealand
7. Older Person’s Health, Canterbury District Health Board, Christchurch, New Zealand

**Corresponding author*:

Professor Philip Schluter

School of Health Sciences

University of Canterbury – Te Whare Wānanga o Waitaha

Private Bag 4800

Christchurch 8140 Tel: +64 3 369 3523

NEW ZEALAND Email: philip.schluter@canterbury.ac.nz

*Definition of demographic and potentially confounding measures*

Age was derived from the difference between date of birth and date of interview. Sex response options were: male, female, unknown, and indeterminate; responses to the last two options were set to missing. Participants are able to self-identify up to a maximum of three ethnic groups. Here, ethnicity was coded using a single priority classification for those with multiple identifications[^1^](#_ENREF_1), with Māori having priority coding, followed by Pacific, Asian, European and Other. Marital status was elicited with six response options: never married; married/civil union/de facto; widowed; separated; divorced; other. For these analyses, separated and divorced categories were combined. Living arrangements at the time of the assessment had response options: alone, with spouse/partner only, with spouse/partner and other(s), with child (not spouse/partner), with parent(s) or guardian(s), with sibling(s), with relatives, and with non-relative(s). Body mass index (BMI) was derived from assessed height and weight measurements (calculated as kg/m^2^) and categorized as: underweight (BMI<18.5); normal (18.5≤BMI<25); overweight (25≤BMI<30); and obese (30≤BMI). However, the validity of a significant proportion of weight and height values has been previously questioned[^2^](#_ENREF_2), and so BMI outliers were identified using Tukey’s method[^3^](#_ENREF_3), and these outlying values were categorised as being unknown (along with those having missing BMI values). Participation in activities of daily living (ADL) was assessed using the ADL hierarchy scale[^4^](#_ENREF_4), which combines four items (personal hygiene, toilet transfer, locomotion, and eating) to create a seven-category score: independence; supervision; limited; extensive; maximal; dependent; and, total dependence. For this scale, the latter two responses were combined and labelled “dependent +”. Instrumental activities of daily living (IADL) combines eight items (meal preparation; ordinary housework; managing finances; managing medications; phone use; stairs; shopping; and, transportation) to derive a score ranging from 0 (complete independence) to 48 (total dependence)[^5^](#_ENREF_5). For the purpose of these analyses, phone use was removed, as it is a marker of hearing loss, and the remaining IADL scores were summed and separated into four approximately equalled sized groups, partitioned around their empirical quartile values. Alcohol consumption found from the highest number of drinks reported in any “single sitting” in the last 14 days, categorized as: none; one; 2-4; 5 or more. For these analyses the last two categories were combined and labelled “2 or more”. Smoking status was ascertained from reported tobacco daily consumption: no; not in last 3 days but usually a smoker; and, yes. The last two categories were combined to define “smoker”. Vision was assessed by asking participants ability to see in adequate light (with glasses or with other visual aid normally used), with response options: adequate – sees fine detail, including regular print in newspapers/books; minimal difficulty – sees large print, but not regular print in newspapers/books; moderate difficulty – limited vision, not able to see newspaper headlines but can identify objects; severe difficulty – object identification in question, but eyes appear to follow objects, sees only light, colours, shapes; and, no vision. The final two categories were combined to form a “severe +” category. Depression indication within the last 3 days employed that Depression Rating Scale[^6^](#_ENREF_6), created by summing seven items (made negative statements; persistent anger with self or others; expressions (including nonverbal) of what appear to be unrealistic fears; repetitive health complaints; repetitive anxious complaints/concerns (non-health-related); sad, pained, worried facial expression; and, crying, tearfulness) and forms a score that ranges from 0 to 14. A score of 0 was labelled “none”; scores 1 and 2 combined and labelled “mild”; and, scores 3-14 combined and labelled “indicated” – in accordance with the DRS guidelines. A delirium screening score was created which combined four items (acute change in mental status from person’s usual functioning; mental function varies over the course of the day; episode of disorganised speech; and, easily distracted)[^7^](#_ENREF_7). A dichotomous score was created, values ≥2 indicating delirium, and values <2 taken as normal; as recommended by its developers. History of falls was elicited from one question, with response options: no falls in last 90 days; no falls in last 30 days, but fell 31-90 days ago; one fall in last 30 days; and, two or more falls in last 30 days. Time since last hospital stay was elicited in one question and coded for the most recent instance in the last 90 days, with six response options: no hospitalisations within 90 days ago; 31-90 days ago; 15-30 days ago; 8-14 days ago; in the last 7 days; and, now in hospital. In this analysis, these options were collapsed into four groups, combining the: 15-30 days ago; 8-14 days ago; and, in the last 7 days options. Disease diagnosis for conditions (including: stroke/cerebrovascular accident (CVA), chronic obstructive pulmonary disease (COPD), cancer, congestive heart failure (CHF), and coronary heart disease (CHD)) were assessed by individual questions following the preamble “Diseases/infections that doctor has indicated is present and affects client’s status, requires treatment, or symptom management. Also include if disease is monitored by a home care professional or is the reason for hospitalization in last 90 days (or since last assessment if less than 90 days), with response options: not present; primary diagnosis/diagnoses for current stay; diagnosis present, receiving active treatment; and, diagnosis, monitored but no active treatment. Here, diseases outcomes were collapsed into binary categories: “not present” and “present”. Fatigue assessed participants’ inability to complete normal daily activities, with response options: none; minimal – diminished energy but completes normal day-to-day activities; moderate – due to diminished energy, unable to finish normal day-to-day activities; severe – due to diminished energy, unable to start some normal day-to-day activities; and, unable to commence any normal day-to-day activities – due to diminished energy. For the purpose of this study, the latter two response options were combined to form a “severe +” category. Timed 4 metre walk was objectively assessed, using a stopwatch (or counting) and the time entered, up until 30 seconds. A code of 30 is recorded for times of 30 or more seconds; 77 if stopped before test complete; 88 if refused to do the test; and, 99 if not tested (e.g. does not walk on own). Here, the last three categories were combined, and the measured times were spilt into approximately tertiles. Urinary incontinence was elicited by asking about incontinent episodes and control over the last three days, with response options: continent – complete control, does not use any type of catheter or urinary collection device; continent with catheter – control with any catheter or ostomy over the last 3 days; infrequently incontinent – not incontinent over last 3 days, but does have incontinent episodes; occasionally incontinent – less than daily; frequently incontinent – daily, but some control present; incontinent – no control present; and, no urine output from bladder in last 3 days. Contiguous response options were collapsed into four categories, namely: “continent”; “infrequently or occasionally incontinent”; “frequently incontinent or incontinent”; and “uses a device/unknown”. Finally, the self-reported health question asked participants, in general, how would they rate their health; with response options: excellent; good; fair; poor; and, could not (would not) response.

*Competing-risk regression model results*

Table S1. Distribution of the demographic and potentially confounding variables for eligible participants, and results of unadjusted and adjusted (using complete cases n=33,993; 99.2%) competing-risk regression analyses relating these variables to aged residential care (ARC) admission.

|  |  | Participants | | | Unadjusted | | Adjusted | |
| --- | --- | --- | --- | --- | --- | --- | --- | --- |
|  |  | n | | (%) | SHR | (95% CI) | SHR | (95% CI) |
| *Age group (years)* | |  | |  |  |  |  |  |
|  | 65-74 | 5,804 | | (16.9) | 1 | (reference) | 1 | (reference) |
|  | 75-84 | 14,323 | | (41.8) | 1.39 | (1.28, 1.50) | 1.27 | (1.17, 1.38) |
|  | 85-94 | 13,088 | | (38.2) | 1.75 | (1.61, 1.89) | 1.39 | (1.27, 1.53) |
|  | 95+ | 1,062 | | (3.1) | 1.81 | (1.57, 2.09) | 1.22 | (1.04, 1.43) |
| *Sex*^a^ | |  | |  |  |  |  |  |
|  | Female | 21,334 | | (62.2) | 1 | (reference) | 1 | (reference) |
|  | Male | 12,942 | | (37.8) | 1.02 | (0.97, 1.07) | 1.11 | (1.04, 1.17) |
| *Ethnicity* | |  | |  |  |  |  |  |
|  | European | 30,254 | | (88.3) | 1 | (reference) | 1 | (reference) |
|  | Māori | 1,782 | | (5.2) | 0.52 | (0.45, 0.61) | 0.55 | (0.47, 0.64) |
|  | Pacific | 1,214 | | (3.5) | 0.38 | (0.31, 0.47) | 0.37 | (0.30, 0.46) |
|  | Other | 1,027 | | (3.0) | 0.65 | (0.55, 0.77) | 0.59 | (0.49, 0.71) |
| *Marital status* | |  | |  |  |  |  |  |
|  | Married/civil union/de facto | 13,519 | | (39.4) | 1 | (reference) | 1 | (reference) |
|  | Widowed | 16,794 | | (49.0) | 1.11 | (1.05, 1.17) | 0.94 | (0.84, 1.06) |
|  | Divorced/separated | 2,330 | | (6.8) | 1.11 | (1.00, 1.22) | 1.19 | (1.03, 1.38) |
|  | Never married | 1,388 | | (4.0) | 1.21 | (1.07, 1.37) | 1.12 | (0.95, 1.32) |
|  | Other | 246 | | (0.7) | 0.95 | (0.68, 1.31) | 0.97 | (0.68, 1.38) |
| *Residential arrangements: living with*^a^ | | | |  |  |  |  |  |
|  | Spouse/partner only | 10,893 | | (31.8) | 1 | (reference) | 1 | (reference) |
|  | Spouse/partner and other(s) | 1,237 | | (3.6) | 0.79 | (0.68, 0.92) | 0.89 | (0.76, 1.04) |
|  | Alone | 16,606 | | (48.4) | 1.13 | (1.07, 1.19) | 1.45 | (1.29, 1.64) |
|  | Child (not spouse/partner) | 3,625 | | (10.6) | 0.96 | (0.88, 1.05) | 1.01 | (0.88, 1.17) |
|  | Other relative(s) | 847 | | (2.5) | 0.90 | (0.75, 1.07) | 1.03 | (0.83, 1.27) |
|  | Non-relative(s) | 1,068 | | (3.1) | 1.75 | (1.54, 1.98) | 1.38 | (1.17, 1.63) |
| *BMI categories* | |  | |  |  |  |  |  |
|  | Underweight | 1,687 | | (4.9) | 1.12 | (1.00, 1.25) | 1.04 | (0.92, 1.16) |
|  | Normal | 9,494 | | (27.7) | 1 | (reference) | 1 | (reference) |
|  | Overweight | 5,967 | | (17.4) | 0.80 | (0.74, 0.86) | 0.87 | (0.81, 0.94) |
|  | Obese | 3,975 | | (11.6) | 0.60 | (0.55, 0.67) | 0.75 | (0.67, 0.83) |
|  | Unknown | 13,154 | | (38.4) | 0.96 | (0.90, 1.01) | 0.96 | (0.90, 1.02) |
| *Activities of daily living (ADL) dependence*^b^ | | | |  |  |  |  |  |
|  | Independent | 20,876 | | (61.4) | 1 | (reference) | 1 | (reference) |
|  | Supervision | 4,775 | | (14.0) | 1.93 | (1.81, 2.06) | 1.33 | (1.24, 1.43) |
|  | Limited | 3,677 | | (10.8) | 1.69 | (1.56, 1.82) | 1.18 | (1.09, 1.28) |
|  | Extensive | 2,395 | | (7.0) | 1.74 | (1.60, 1.91) | 1.16 | (1.05, 1.28) |
|  | Maximal | 953 | | (2.8) | 1.55 | (1.34, 1.78) | 0.90 | (0.76, 1.06) |
|  | Dependent + | 1,325 | | (3.9) | 1.27 | (1.11, 1.45) | 0.83 | (0.71, 0.97) |
| *Instrumental activities of daily living (IADL) score*^c^ | | | | |  |  |  |  |
|  | Q_1_ (0-14) | 8,310 | | (24.4) | 1 | (reference) | 1 | (reference) |
|  | Q_2_ (15-24) | 8,326 | | (24.5) | 1.80 | (1.65, 1.97) | 1.81 | (1.65, 1.98) |
|  | Q_3_ (25-33) | 8,652 | | (25.4) | 2.57 | (2.36, 2.79) | 2.60 | (2.37, 2.85) |
|  | Q_4_ (34-42) | 8,714 | | (25.6) | 3.18 | (2.93, 3.46) | 3.25 | (2.93, 3.60) |
| *Alcohol consumption (drinks)*^a^ | |  | |  |  |  |  |  |
|  | None | 26,875 | | (78.4) | 1 | (reference) | 1 | (reference) |
|  | 1 | 4,688 | | (13.7) | 0.85 | (0.79, 0.92) | 0.88 | (0.81, 0.95) |
|  | 2+ | 2,713 | | (7.9) | 0.81 | (0.73, 0.89) | 0.87 | (0.78, 0.96) |
| *Smoking status*^d^ | |  | |  |  |  |  |  |
|  | Non-smoker | 32,491 | | (94.8) | 1 | (reference) | 1 | (reference) |
|  | Smoker | 1,783 | | (5.2) | 1.00 | (0.89, 1.11) | 1.08 | (0.96, 1.21) |
| *Visual impairment*^d^ | |  | |  |  |  |  |  |
|  | None | 24,399 | | (71.2) | 1 | (reference) | 1 | (reference) |
|  | Minimal | 6,639 | | (19.4) | 1.27 | (1.19, 1.35) | 1.11 | (1.04, 1.18) |
|  | Moderate | 2,319 | | (6.8) | 1.37 | (1.25, 1.50) | 1.06 | (0.96, 1.16) |
|  | Severe + | 917 | | (2.7) | 1.21 | (1.04, 1.39) | 0.87 | (0.75, 1.01) |
| *Depression status*^b^ | |  | |  |  |  |  |  |
|  | None | 21,591 | | (63.5) | 1 | (reference) | 1 | (reference) |
|  | Mild | 7,759 | | (22.8) | 1.31 | (1.24, 1.39) | 1.22 | (1.15, 1.30) |
|  | Indicated | 4,651 | | (13.7) | 1.48 | (1.39, 1.59) | 1.35 | (1.26, 1.46) |
| *Delirium* | |  | |  |  |  |  |  |
|  | None | 32,071 | | (93.6) | 1 | (reference) | 1 | (reference) |
|  | Indicated | 2,206 | | (6.4) | 1.68 | (1.54, 1.83) | 1.25 | (1.14, 1.37) |
| *History of falls*^a^ | |  | |  |  |  |  |  |
|  | No falls in last 90 days | 21,415 | | (62.5) | 1 | (reference) | 1 | (reference) |
|  | Fell 31-90 days ago | 3,841 | | (11.2) | 1.16 | (1.07, 1.25) | 1.04 | (0.96, 1.12) |
|  | One fall in last 30 days | 5,403 | | (15.8) | 1.29 | (1.20, 1.37) | 1.08 | (1.01, 1.16) |
|  | Two or more falls in last 30 days | 3,617 | | (10.6) | 1.56 | (1.45, 1.68) | 1.21 | (1.12, 1.31) |
| *Most recent hospitalisation* | |  | |  |  |  |  |  |
|  | None within last 90 days | 21,007 | | (61.3) | 1 | (reference) | 1 | (reference) |
|  | 31-90 days ago | 4,881 | | (14.2) | 0.88 | (0.81, 0.95) | 0.88 | (0.81, 0.95) |
|  | In the last 30 days | 4,167 | | (12.2) | 1.02 | (0.95, 1.11) | 0.99 | (0.91, 1.07) |
|  | Now in hospital | 4,222 | | (12.3) | 1.41 | (1.31, 1.51) | 1.04 | (0.96, 1.13) |
| *Stroke/CVA* | |  | |  |  |  |  |  |
|  | Not present | 28,439 | | (83.0) | 1 | (reference) | 1 | (reference) |
|  | Diagnosed | 5,838 | | (17.0) | 0.99 | (0.93, 1.05) | 0.91 | (0.85, 0.97) |
| *Chronic obstructive pulmonary disease* | | |  |  |  |  |  |  |
|  | Not present | 28,836 | | (84.1) | 1 | (reference) | 1 | (reference) |
|  | Diagnosed | 5,441 | | (15.9) | 0.85 | (0.80, 0.92) | 0.98 | (0.91, 1.05) |
| *Cancer* | |  | |  |  |  |  |  |
|  | Not present | 29,977 | | (87.5) | 1 | (reference) | 1 | (reference) |
|  | Diagnosed | 4,300 | | (12.5) | 0.85 | (0.79, 0.92) | 0.86 | (0.80, 0.93) |
| *Fatigue*^a^ | |  | |  |  |  |  |  |
|  | None | 10,400 | | (30.3) | 1 | (reference) | 1 | (reference) |
|  | Minimal | 11,722 | | (34.2) | 0.87 | (0.82, 0.93) | 0.87 | (0.82, 0.93) |
|  | Moderate | 8,368 | | (24.4) | 0.94 | (0.88, 1.01) | 0.85 | (0.79, 0.91) |
|  | Severe + | 3,786 | | (11.0) | 0.90 | (0.83, 0.99) | 0.71 | (0.65, 0.79) |
| *Congestive heart failure* | |  | |  |  |  |  |  |
|  | Not present | 28,266 | | (82.5) | 1 | (reference) | 1 | (reference) |
|  | Diagnosed | 6,011 | | (17.5) | 0.91 | (0.85, 0.97) | 0.90 | (0.84, 0.96) |
| *Coronary heart disease* | |  | |  |  |  |  |  |
|  | Not present | 23,023 | | (67.2) | 1 | (reference) | 1 | (reference) |
|  | Diagnosed | 11,254 | | (32.8) | 0.97 | (0.92, 1.02) | 1.02 | (0.97, 1.08) |
| *Timed 4 metre walk (seconds)* | |  | |  |  |  |  |  |
|  | ≤8 | 10,383 | | (30.3) | 1 | (reference) | 1 | (reference) |
|  | 9-14 | 8,490 | | (24.8) | 1.07 | (1.00, 1.14) | 0.96 | (0.90, 1.03) |
|  | ≥15 | 8,760 | | (25.6) | 1.15 | (1.08, 1.23) | 0.96 | (0.89, 1.02) |
|  | Incomplete/refused/not tested | 6,644 | | (19.4) | 1.19 | (1.11, 1.28) | 0.89 | (0.82, 0.97) |
| *Urinary incontinence* | |  | |  |  |  |  |  |
|  | Continent | 20,767 | | (60.6) | 1 | (reference) | 1 | (reference) |
|  | Infreq./occasionally incontinent | 6,042 | | (17.6) | 1.32 | (1.24, 1.41) | 1.14 | (1.06, 1.22) |
|  | Freq. incontinent/incontinent | 5,879 | | (17.2) | 1.42 | (1.33, 1.51) | 1.19 | (1.11, 1.27) |
|  | Uses a device/unknown | 1,589 | | (4.6) | 1.22 | (1.09, 1.36) | 0.99 | (0.88, 1.12) |
| *Self-rated health* | |  | |  |  |  |  |  |
|  | Excellent | 1,094 | | (3.2) | 1 | (reference) | 1 | (reference) |
|  | Good | 14,550 | | (42.4) | 0.92 | (0.81, 1.06) | 0.91 | (0.79, 1.05) |
|  | Fair | 12,412 | | (36.2) | 0.85 | (0.74, 0.98) | 0.84 | (0.73, 0.97) |
|  | Poor | 3,715 | | (10.8) | 0.91 | (0.79, 1.06) | 0.86 | (0.73, 1.01) |
|  | No response | 2,506 | | (7.3) | 1.39 | (1.19, 1.62) | 1.02 | (0.87, 1.21) |

Note: ^a^1 observation missing; ^b^276 observations missing; ^c^275 observations missing; ^d^3 observations missing.

**References**

1. Allan, J.-A. Review of the Measurement of Ethnicity: Classification and Issues. (Statistics New Zealand, Wellington, 2001).
2. Schluter, P. J. *et al*. Comprehensive clinical assessment of home-based older persons within New Zealand: an epidemiological profile of a national cross-section. *Aust N Z J Public Health* **40**, 349-355 (2016).
3. Tukey, J. W. Exploratory Data Analysis. (Addison-Wesley Reading, PA, 1999).
4. Morris, J. N., Fries, B. E. & Morris, S. A. Scaling ADLs within the MDS. *J Gerontol A Biol Sci Med Sci* **54**, M546-553 (1999).
5. Morris, J. N. *et al*. Scaling functional status within the interRAI suite of assessment instruments. *BMC Geriatr* **13**, 128 (2013).
6. Burrows, A. B. *et al*. Development of a minimum data set-based depression rating scale for use in nursing homes. *Age Ageing* **29**, 165-172 (2000).
7. Salih, S. A. *et al*. Screening for delirium within the interRAI acute care assessment system. *J Nutr Health Aging* **16**, 695-700 (2012).
